# Monocyte Trajectories Endotypes Are Associated With Worsening in Septic Patients

**DOI:** 10.3389/fimmu.2021.795052

**Published:** 2021-11-29

**Authors:** Maxime Bodinier, Estelle Peronnet, Karen Brengel-Pesce, Filippo Conti, Thomas Rimmelé, Julien Textoris, Christophe Vedrine, Laurence Quemeneur, Andrew D. Griffiths, Lionel K. Tan, Fabienne Venet, Delphine Maucort-Boulch, Guillaume Monneret, the REALISM study group

**Affiliations:** ^1^ EA 7426 “Pathophysiology of Injury-Induced Immunosuppression” (Université Claude Bernard Lyon 1 - Hospices Civils de Lyon - bioMérieux), Joint Research Unit HCL-bioMérieux, Immunology Laboratory & Anesthesia and Critical Care Medicine Department, Hospices Civils de Lyon, Edouard Herriot Hospital, Lyon, France; ^2^ BIOASTER Technology Research Institute, Bioassays, Microsystems and Advanced Optics Engineering Unit, Lyon, France; ^3^ Sanofi Pasteur, Human Immunology Unit, Marcy l’Etoile, France; ^4^ Laboratoire de Biochimie (LBC), ESPCI Paris, PSL Université, CNRS UMR8231, Paris, France; ^5^ GlaxoSmithKline (GSK), Clinical Development Unit, Brentford, United Kingdom; ^6^ Centre International de Recherche en Infectiologie (CIRI), Inserm U1111, CNRS, UMR5308, Ecole Normale Supérieure de Lyon, Université Claude, Lyon, France; ^7^ Université Claude Bernard Lyon 1, Université de Lyon, Lyon, France; ^8^ Équipe Biostatistique-Santé, Laboratoire de Biométrie et Biologie Évolutive, CNRS UMR 5558, Villeurbanne, France; ^9^ Service de Biostatistique-Bioinformatique, Pôle Santé Publique, Hospices Civils de Lyon, Lyon, France

**Keywords:** sepsis, endotype, trajectory, immune monitoring, ICU– Intensive Care Unit, monocyte HLA-DR, flow cytometry, immunosuppression

## Abstract

Sepsis is a life-threatening organ dysfunction caused by a dysregulated host response to infection. The immune system plays a key role in sepsis onset and remains dysregulated over time in a heterogeneous manner. Here, we decipher the heterogeneity of the first week evolution of the monocyte HLA-DR (mHLA-DR) surface protein expression in septic patients, a key molecule for adaptive immunity onset. We found and verified four distinctive trajectories endotypes in a discovery (n = 276) and a verification cohort (n = 102). We highlight that 59% of septic patients exhibit low or decreasing mHLA-DR expression while in others mHLA-DR expression increased. This study depicts the first week behavior of mHLA-DR over time after sepsis onset and shows that initial and third day mHLA-DR expression measurements is sufficient for an early risk stratification of sepsis patients. These patients might benefit from immunomodulatory treatment to improve outcomes. Going further, our study introduces a way of deciphering heterogeneity of immune system after sepsis onset which is a first step to reach a more comprehensive landscape of sepsis.

## Introduction

Sepsis is defined as a life-threatening organ dysfunction due to a dysregulated host immune response to infection (1). This defines the onset of sepsis and the immune response remains dysregulated afterwards with two parallel avenues, hyper-inflammation and immunosuppression. This post-sepsis immune response is heterogeneous and several studies have attempted to define this heterogeneity at admission to the Intensive Care Unit (ICU) (2–6). However, little is known about the heterogeneity in the dynamics of immune markers over time, or the immune trajectories of individual patients. One method to understand this longitudinal heterogeneity is to break down the dataset into more homogeneous groups, or trajectory endotypes. Subsequently, this could lead to a better understanding of the pathophysiology of sepsis and help to identify associations between endotypes and poor clinical outcomes.

The post-sepsis hyper-inflammatory response is compensated by an immunosuppressive response. In some cases, this immunosuppressive response may continue in spite of the pro-inflammatory response returning to normal. This persistent immunosuppression could predispose to nosocomial infections, with longer ICU stays and increased mortality as consequences. The monocyte human leukocyte antigen DR surface protein (mHLA-DR) plays a key role in the onset of the adaptive immune response by enabling antigen presentation to T cells. A decrease in mHLA-DR has been shown to be associated with poor clinical outcomes (7–11) and thus was proposed to be a marker of an immunosuppressive response. In preliminary work, Leijte et al. highlighted the possible utility of mHLA-DR trajectory endotyping for determining clinical outcome prognosis. However, the method used didn’t tolerate missing values and thus patient with missing time points were dropped, resulting in a smaller cohort with a patient selection bias. Moreover, the reproducibility of mHLA-DR endotypes and the association with clinical outcomes was not studied.

Here, we aimed to accurately identify mHLA-DR trajectory endotypes in sepsis patients during the first week after ICU admission. We applied a trajectory clustering method, K-means for longitudinal data – KmL (12), to a discovery cohort; this methodology enables handling of missing data in longitudinal studies. We then confirmed our observations in an independent verification cohort and evaluated the identified endotypes in relation to the occurrence of ICU Acquired Infection (IAI), death and ICU discharge over the first 28 days after inclusion.

## Materials and Methods

### Patient Cohorts

Two observational cohorts were used for the current study. A Discovery cohort from the IMMUNOSEPSIS study (13) that included 333 patients admitted to Lyon University Hospital, France from March 2014 to July 2018. This prospective study aimed to characterize Major Histocompatibility Complex (MHC) class II expression on monocytes in septic shock patients and evaluated its association with ICU-acquired infection. Study patients were screened for septic shock based on Sepsis-2 criteria before 2016 and Sepsis-3 criteria (1) after 2016. To comply with Sepsis-3 definitions, patients with lactate ≤ 2 mmol/L at admission were labelled as sepsis patients (n=83). Data were collected at three time points during the first week after study inclusion: D1 or D2, D3 or D4 and D5, D6 or D7. The IMMUNOSEPSIS cohort is registered with the French Ministry of Research and Teaching (#DC-2008-509), at the Commission Nationale de l’Informatique et des Libertés, at Clinicaltrials.gov (NCT02803346) and has been approved by the Institutional Study Board (IRB11236).

The verification cohort was from the REALISM study (14), which aimed to characterize and follow up injury-induced immunosuppression in critically ill patients. This study recruited patients admitted to Lyon University Hospital, France from December 2015 to June 2018; 107 sepsis patients met the Sepsis-3 criteria (Sepsis n=31, Septic Shock n=76). The study design has been extensively reviewed in Rol et al. (15) and will not be repeated in detail here. Data were collected at three time points during the first week after study inclusion: D1 or D2, D3 or D4 and D5, D6 or D7. The REALISM study is registered at ClinicalTrials.gov (NCT02638779) and have been approved by the Institutional Study Board (2015-42-2).

### Monocytic HLA-DR Measurement

For the two cohorts, the mHLA-DR expression was quantified as the number of antibodies bound per monocyte (AB/C). This was achieved on whole blood by standardized flow cytometry using the Anti-HLA-DR/Anti-Monocytes Quantibrite assay (BD Biosciences, San Jose, USA) and described elsewhere (16, 17).

### Outcomes

The main clinical outcomes included here are ICU Acquired infection (IAI), death and ICU discharge over the first 28 days after study inclusion. For the REALISM study, information related to infections were collected, reviewed and validated by a blinded dedicated adjudication committee, composed of three physicians with confirmation of secondary infection made according to the definitions used by the European Centre for Disease Prevention and Control and the Infectious Diseases Society of America. For the IMMUNOSEPSIS study, IAI were collected, reviewed and validated by an intensivist according to definitions proposed by the committee for nosocomial infections and healthcare associated infections (CNTILS) of the French Ministry of Health.

### Statistical Analysis

#### Unsupervised Clustering

In order to identify patients’ group with a common mHLA-DR dynamic over time (trajectories endotypes) we used KmL – K-means for Longitudinal data – R package 2.4.1 (12). The number of endotypes was *a priori* defined based on the literature (18). A boxcox transformation was applied to the mHLA-DR marker to normalize the distribution before outlier samples detection and exclusion based on Tukey method (19). The KmL method pipeline works as follow: first marker’s trajectories are clustered using k-means algorithm with a Gower adjusted Euclidean distance metric to handle missing data. Secondly, Calinski-Harabasz metric is studied to compare the different partitions; this represents the ratio between within-cluster and between-cluster dispersion and is thus a surrogate of partitioning quality. Within each cluster, missing values are imputed using linear interpolation so that imputed values follow the population mean trajectory shape. In order to avoid convergence to local minimum, the KmL method was run a thousand times, selecting the run showing the highest Calinski-Harabasz metric. Mixed effect model was fitted to mHLA-DR data using R package lme4 1.1-26 (20) to highlight mean mHLA-DR tendencies per cluster. A boxcox transformation was applied to the mHLA-DR marker to enable linear modeling. The model sets a second-degree polynomial fixed effect for time per endotype and a random intercept and slope of time to account for correlation between patient’s observations. Around mean curve, 95% confidence intervals were computed using R package merTools 0.5.2 (21).

#### Trajectories Comparison Between Cohorts

The mean trajectories of endotypes from both cohorts were compared using a t-test with degree of freedom adjusted for mixed models [R package lmerTest 3.1.3 (22)].

#### Clinical Characterization of Endotypes

The clinical characteristics of the cohorts were described by endotype. Continuous data were summarized by median, inter quartile range, mean and standard deviation. Categorical data were summarized by sample sizes and percentages. Endotypes were compared either with analysis of variance (ANOVA) test in case of normally distributed data or with Kruskal Wallis test by ranks for continuous data Chi-squared tests or Fisher’s exact tests were used for categorical data.

#### Outcomes Characterization of Endotypes

The probabilities of different outcomes per endotype were estimated using a competing risk analysis. Discharge from ICU and death were considered to be competing outcomes of an IAI event. The probability of each of these events up to the last remaining patient at risk was graphically reported. To evaluate the impact on outcome of different endotypes, we extracted sub-distribution Hazard Ratios from a Fine-Gray model (23) with the same setting of competing risks.

Normality of data distribution was assessed using Shapiro-Wilk tests. The null hypothesis was rejected for a p value less than 0.01. All statistical analysis and visualizations were done using R 3.6.2 (24). All graphics were done using ggplot v3.3.2 (25).

## Results

### Cohort Characteristics

To assess trajectory endotypes, we selected 276 patients from Discovery cohort and 102 patients in the Verification cohort (**sFigure 1**). Overall characteristics of the patients included in the cohorts are summarized in **Table 1**. Both cohorts were similar, despite a higher SAPS II score, a higher use of vasopressors in the discovery cohort and a higher us of RRT in the verification cohort. Verification cohort exhibited a higher proportion of community acquired infections as source of sepsis. This translated in a higher mortality and IAI rates among patients in the discovery cohort.

**Table 1:** Cohort characteristics.

	Discovery cohort n = 276	Verification cohort n = 102	p.	Aggregated dataset n = 378
**Shock at inclusion**	200 (72.5%)	72 (70.6%)	0.817	272 (72.1%)
**Infection Acquisition Type**				
*Community acquired*	152 (55.1%)	72 (71.0%)	**0.009**	224 (59.0%)
*Hospital acquired*	124 (45.0%)	30 (29.4%)		154 (40.8%)
**Primary infection location**				
*Abdominal*	145 (52.5%)	51 (50.0%)	0.718	196 (51.9%)
*Catheter and/or bacteremia*	10 (3.6%)	2 (2.0%)		12 (3.2%)
*Other*	64 (23.2%)	23 (22.6%)		87 (23.0%)
*Respiratory*	57 (20.7%)	26 (25.5%)		83 (22.0%)
**Age (year)**	71 [63-79]	68 [59-78]	0.087	70 [62-79]
**Gender (Female)**	94 (34.1%)	36 (35.3%)	0.918	130 (34.4%)
**McCabe**				
*Non-fatal disease*	138 (50.2%)	62 (60.8%)	0.096	200 (53.1%)
*Rapidly fatal disease(within 1 year)*	33 (12.0%)	6 (5.9%)		39 (10.3%)
*Ultimately fatal disease(within 5 years)*	104 (37.8%)	34 (33.3%)		138 (36.6%)
**Charlson score**	2.0 [1.0-4.0]	2.0 [1.0-3.0]	0.189	2.0 [1.0-4.0]
**SAPS II score**	58.0 [49.0-70.8]	46.5 [37.0-54.0]	**<0.001**	55.0 [45.0-66.3]
**SOFA score D1**	9.0 [7.0-11.0]	9.0 [7.0-11.0]	0.335	9.0 [7.0-11.0]
**D30 Urine Catheter free days**	21 [1-25]	17 [0-26]	**-**	20 [1-25]
**D30 Venous Catheter free days**	26 [10-28]	13 [0-22]	**-**	24 [7-28]
**D30 Intubation free days**	24 [9-29]	26 [9-29]	**-**	25 [9-29]
**Renal replacement therapy**	61 (22.1%)	38 (37.3%)	**0.004**	99 (26.2%)
**Coma (GCS <8)**	29 (10.7%)	6 (5.9%)	0.221	35 (9.4%)
**Vasopressors**	276 (100.0%)	95 (93.1%)	**<0.001**	371 (98.2%)
**D30 Hospital free days**	0 [0-9]	0 [0-12]	**-**	0 [0-10]
**D30 ICU free days**	17 [0-23]	23 [12-26]	**-**	19 [0-24]
**D28 Death**	71 (25.7%)	14 (13.7%)	**-**	85 (22.5%)
**D28 IAI**	50 (18.1%)	10 (9.9%)	**-**	60 (15.9%)

SAPS II, Simplified Acute Physiological Score II; SOFA, Sequential Organ Failure Assessment score; D, Day; MV, mechanical ventilation; ICU, Intensive Care Unit; GCS, Glasgow coma scale; IAI, Intensive Care Unit associated Infection; p., p-value. Data are presented as numbers and percentages (qualitative variables) and medians and 25th/75th percentiles (quantitative variables). Categorical data were summarized by sample sizes and percentages. Cohorts were compared either with analysis of variance (ANOVA) test in case of normally distributed data or with Kruskal Wallis test by ranks for continuous data, and Chi-squared test or Fisher’s exact test, where required, for categorical data. No comparison were done for ‘D30 Urine Catheter free days’, ‘D30 Venous Catheter free days’, ‘D30 Intubation free days’, ‘D30 Hospital free days’, ‘D30 ICU free days’, ‘D28 Death’ and ‘D28 IAI’ as per these data require survival models for comparison (displayed by dash in “p.” column).

### Defining Reliable mHLA-DR Endotypes

**Figure 1:**
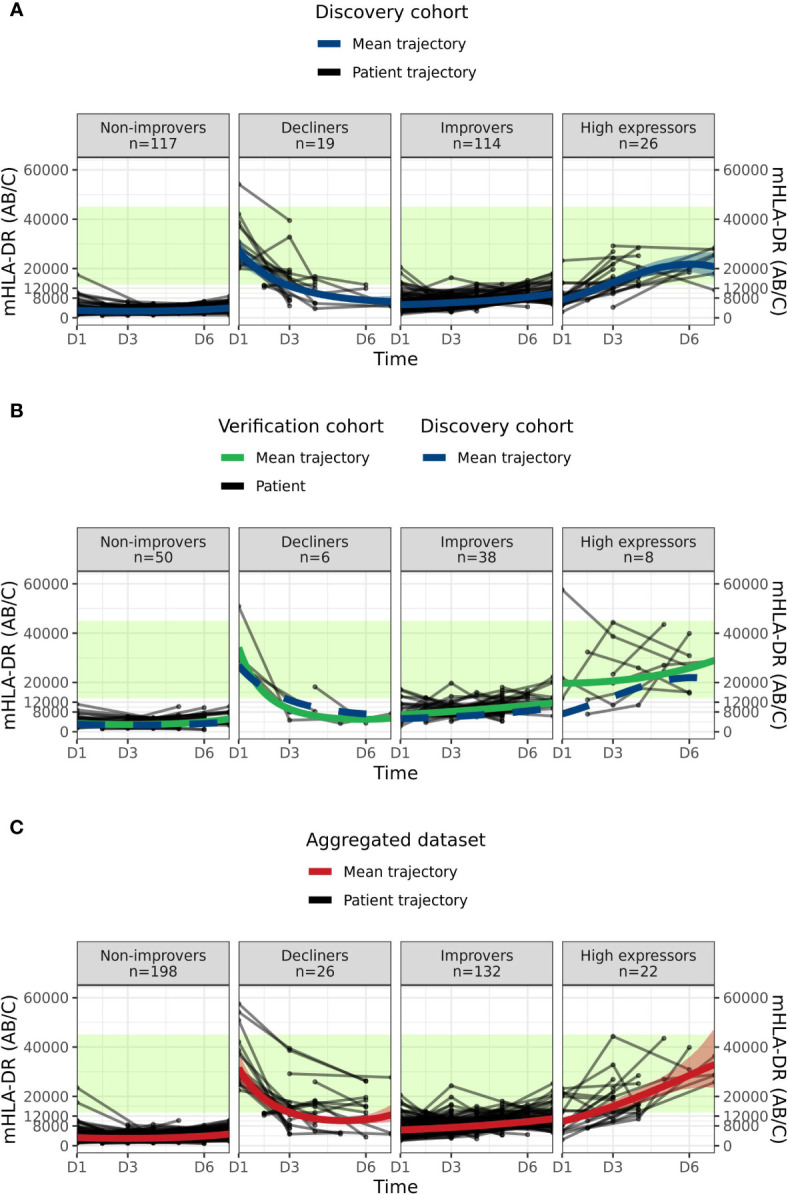
Sepsis mHLA-DR trajectory endotypes. **(A)** Discovery dataset: “Non-improvers”, “Decliners”, “Improvers” and “High expressors” endotypes from KmL clustering method are represented in each panel. Black lines and dots correspond to individual patients mHLA-DR trajectories and values at the sampling time point, respectively. Blue line and blue band represent the mean endotype trajectory from a mixed effect model and the associated 95% confidence interval. The green area represents Healthy Volunteers reference interval, as defined in (14). **(B)** Verification dataset “Non-improvers”, “Decliners”, “Improvers” and “High expressors” endotypes from a clustering *de novo* with KmL method are represented in each panel. Black lines and points respectively correspond to verification cohort’s patients mHLA-DR trajectory and their sampling time point. The green area represents Healthy Volunteers reference interval, has defined in (14). Green solid line and blue dashed line represent the mean trajectory from a mixed effect model of verification cohort and discovery cohort, respectively. **(C)** Discovery and Verification cohorts were merged in an “Aggregated dataset” and KML unsupervised clustering was run *de novo* on mHLA-DR expression values. The figure represents patient trajectories in each panel. Black lines and points correspond to aggregated dataset patients mHLA-DR trajectory and their sampling time point, respectively. Red line and red band represent from a mixed effect model the mean endotype trajectory and the associated 95% confidence interval, respectively. The green area represents Healthy Volunteers reference interval, defined in (14). Mean trend mHLA-DR values are reported in **sTable 3**.

The KmL clustering method resulted in the definition of the following four endotypes in the Discovery cohort: an endotype showing constantly low or barely increasing mHLA-DR expression, with a mean trend always below 4000 AB/C, that we named “Non-improvers”; an endotype starting from mHLA-DR reference interval values and decreasing to below 7500 AB/C, that we named “Decliners”; an endotype showing an increasing mHLA-DR expression that almost reaches the reference interval at the end of the first week, that we named “Improvers”; and an endotype with mHLA-DR rapidly reaching the reference interval, that we named “High expressors” (**Figure 1A**). The mean probability of a patient being assigned to an endotype was always greater than 0.78, which suggests very good quality partitioning and classification using this methodology (**sTable 1**).

Next, to confirm that the observed endotype clusters were not only specific to the Discovery cohort, we applied the same unsupervised clustering method to the Verification cohort. Non-improvers, Decliners, Improvers and High expressor endotypes again were recovered (**Figure 1B**) with very good quality classification (**sTable 1**). We quantitatively verified this observation using a mixed effects model comparing the mean trajectory parameters for each endotype in the Discovery and Verification cohorts (**sTable 2**). We additionally observed that there was no significant difference (p=0.8, Chi-squared test) in the proportion of patients in each endotype between the Discovery and Verification cohorts: Non-improvers (42% vs 49%); Decliners (7% vs 6%); Improvers (41% vs 37%); and High expressors (9% vs 8%).

**Figure 2:**
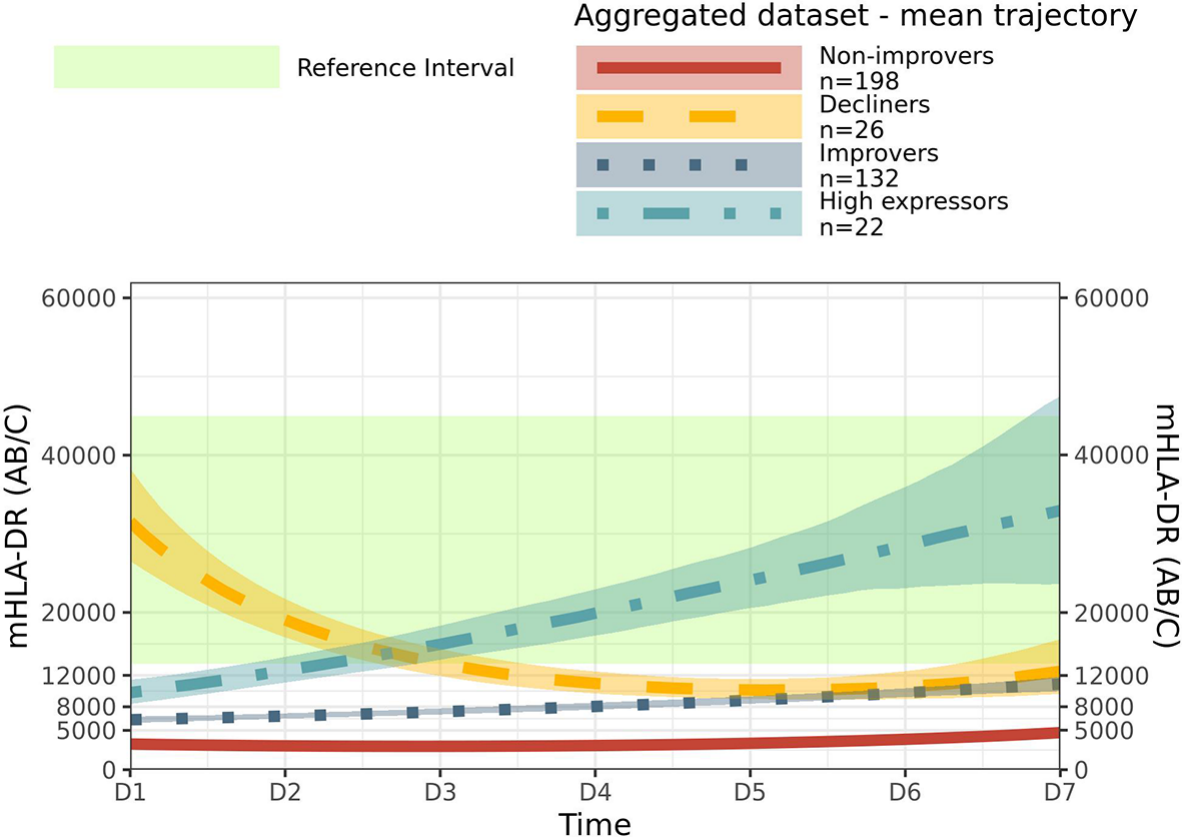
Mean trajectory per endotype in aggregated dataset. Mean trajectory of mHLA-DR endotypes in aggregated dataset were together drawn. Non-Improvers (red, plain line), Decliners (gold, long dashed line), Improvers (dark blue, dotted line) and High expressors (light blue, dashed and dotted line). 95% confidence intervals were drawn as areas around mean trend. The green area represents Healthy Volunteers reference interval, has defined in (14).

As there was no difference between patient proportions in each endotype between cohorts and to increase the number of patients in each endotype to achieve more reliable partitioning and clinical description of endotypes, we aggregated the Discovery and Verification cohorts and applied *de novo* the unsupervised clustering method. The algorithm converged on the same four endotypes with the same trend as previously described: Non-improvers (mean D1 = 3265 AB/C, mean D6 = 3865 AB/C); Decliners improvers (mean D1 = 31,774 AB/C, mean D6 = 10,785 AB/C); Improvers (mean D1 = 6331 AB/C, mean D6 = 9787 AB/C); and High expressors (mean D1 = 9681 AB/C, mean D6 = 28,604 AB/C) (**Figure 1C**, **Figure 2** and **sTable 4**). We observed a balanced repartitioning of Verification cohort patients across all endotypes with mean patient classification probability of greater than 0.75 for each endotype (**sTable 1**). Concordance of 85% was observed for patient matching between Discovery, Verification and Aggregated datasets (**sFigure 2**). Different combinations of sampling time points did not alter the observed clustering (**sFigure 3**). All subsequent endotype-specific clinical characterizations were based on the Aggregated dataset.

### Association Between mHLA-DR Endotypes and Clinical Outcomes in the Aggregated Sepsis Dataset

**Figure 3:**
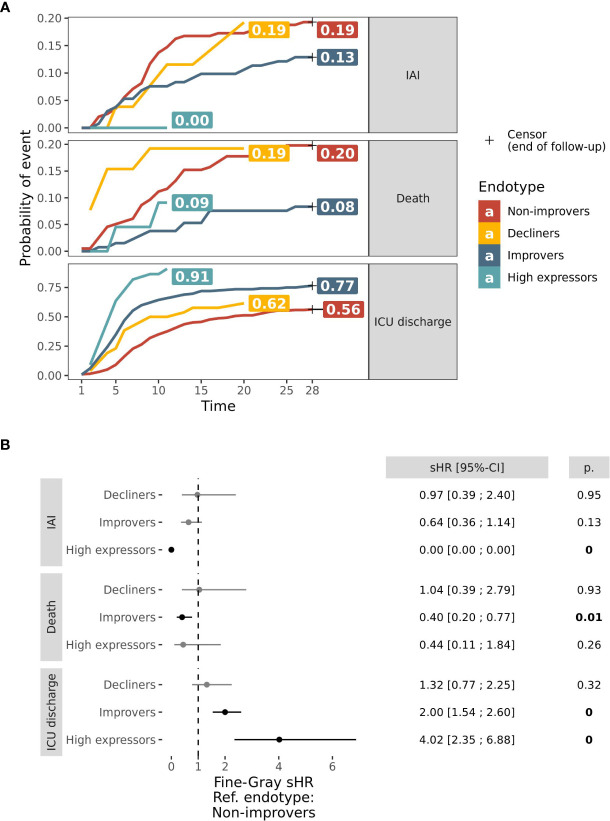
D28 outcomes in mHLA-DR endotypes. **(A)** Curves of probabilities over 28 days of IAI (top pane), Death (middle pane) and ICU discharge (bottom pane) in Aggregated dataset’s endotypes: Non-improvers (red), Decliners (gold), Improvers (dark blue) and High expressors (light blue). These probabilities were estimated through a survival model of IAI with Death and ICU discharge as competing risks and are expressed as cumulated probabilities. Probabilities at the time of the latest remaining patient at risk were reported on the right hand side of curves. **(B)** Forest plot of Fine-Gray regression sub-distribution Hazard Ratio (sHR) of outcome by endotype, in comparison with Non-improvers. sHR were reported graphically (black point) and numerically along with 95% Confidence Interval (CI, horizontal bars). sHR significantly different from 1 were reported in bold and p value (p.) was numerically reported.

Clinical characteristics at inclusion showed no differences across endotypes (**Table 2**): shock at inclusion, infection acquisition type, age, gender, McCabe and Charlson scores were all comparable between endotypes. Although SOFA (p < 0.001) severity score at admission was significantly different, this difference was not clinically relevant, with a median SOFA score ranging from 8 to 9. In contrast, sepsis in the Non-improvers endotype was more often associated with respiratory infections than in other endotypes (28% in Non-improvers, 15% for Decliners, 16% for Improvers and 9% for High expressors). There was a difference between endotypes in outcome measures including the duration of exposure to invasive devices (D30 urinary catheter, venous catheter and intubation free days), hospital and ICU length of stay, death and IAI occurrences. The Non-improvers and Decliners endotypes were similar in outcome measures, with (i) an equal incidence of cases of IAI (0.19 and 0.19 respectively) and death (0.2 and 0.19) (**Figure 3A**) and (ii) no significant difference in the sub-distribution Hazard Ratios (sHR) between these two endotypes (**Figure 3B**). Specifically, these two endotypes appeared to be associated with poorer outcomes, with an increased length of hospital and ICU stay, longer exposure to invasive devices, and increased death and IAI occurrences compared to the Improvers and High expressors endotypes. Additionally, we observed no significant differences in outcome measures between the Non-improvers and Decliners endotype when comparing sub-distribution Hazard Ratio (sHR). Conversely, compared to Non-improvers, Improvers were less prone to death (sHR 0.40 95% CI [0.20; 0.77]) and to have a longer ICU stay (sHR 2.00 [1.54; 2.60]); whilst the High expressors endotype was associated with lower risk of IAI events (sHR 0, because of IAI event absence among the 22 patients) and to have a shorter ICU stay (sHR 4.02 [2.35; 6.88]). After 11 days in ICU, no patient remained at risk in the High expressors endotype, and the incidence of ICU discharge event was 0.91, this was greater than Improvers (0.77), Decliners (0.62) and Non-improvers (0.56) endotypes.

**Table 2:** Aggregated Sepsis cohort mHLA-DR trajectories endotypes characteristics.

	Non-improvers n = 198	Decliners n = 26	Improvers n = 132	High expressors n = 22	p.
**Verification cohort**	48 (24.2%)	8 (30.8%)	41 (31.1%)	5 (22.7%)	0.519
**Shock at inclusion**	151 (76.3%)	15 (57.7%)	92 (69.7%)	14 (63.6%)	0.137
**Infection Acquisition Type**					
*Community acquired*	119 (60.1%)	16 (61.5%)	78 (59.1%)	11 (50.0%)	0.826
*Hospital acquired*	79 (39.9%)	10 (38.5%)	54 (40.9%)	11 (50.0%)	
**Primary infection location**					
*Abdominal*	93 (47.0%)	11 (42.3%)	80 (60.6%)	12 (54.6%)	**<0.001**
*Catheter and/or bacteremia*	0 (0.0%)	5 (19.2%)	5 (3.8%)	2 (9.1%)	
*Other*	49 (24.8%)	6 (23.1%)	26 (19.7%)	6 (27.3%)	
*Respiratory*	56 (28.3%)	4 (15.4%)	21 (15.9%)	2 (9.1%)	
**Age (year)**	69 [62-79]	74 [67-78]	70 [61-78]	68 [59-81]	0.807
**Gender (Female)**	62 (31.3%)	13 (50.0%)	51 (38.6%)	4 (18.2%)	0.066
**McCabe**					
*Non-fatal disease*	115 (58.4%)	10 (38.5%)	65 (49.2%)	10 (45.5%)	0.263
*Rapidly fatal disease(within 1 year)*	17 (8.6%)	2 (7.7%)	17 (12.9%)	3 (13.6%)	
*Ultimately fatal disease(within 5 years)*	65 (33.0%)	14 (53.9%)	50 (37.9%)	9 (40.9%)	
**Charlson score**	2.0 [1.0-3.0]	3.0 [1.0-4.0]	2.0 [1.0-4.0]	3.0 [1.3-4.8]	0.062
**SAPS II score**	57.5 [47.0-67.8]	54.0 [42.5-74.3]	52.5 [43.3-63.8]	49.5 [41.3-63.0]	0.047
**SOFA score D1**	9.0 [8.0-12.0]	9.0 [8.0-11.0]	8.0 [6.0-10.0]	8.0 [7.0-9.0]	**<0.001**
**D30 Urine Catheter free days**	17 [0-23]	17 [1-25]	23 [7-27]	26 [24-29]	**-**
**D30 Venous Catheter free days**	22 [2-27]	21 [5-27]	25 [13-28]	28 [25-29]	**-**
**D30 Intub. free days**	23 [3-27]	22 [8-30]	27 [18-29]	29 [28-30]	**-**
**Renal replacement therapy**	60 (30.3%)	10 (38.5%)	21 (15.9%)	8 (36.4%)	**0.007**
**Coma (GCS <8)**	21 (10.8%)	4 (15.4%)	9 (6.9%)	1 (4.6%)	0.393
**Vasopressors**	196 (99.0%)	26 (100.0%)	127 (96.2%)	22 (100.0%)	0.294
**D30 Hospital free days**	0 [0-4]	0 [0-9]	1 [0-16]	11 [4-19]	**-**
**D30 ICU free days**	12 [0-22]	17 [4-24]	23 [11-25]	25 [23-27]	**-**
**D28 Death**	55 (27.8%)	6 (23.1%)	22 (16.7%)	2 (9.1%)	**-**
**D28 IAI**	38 (19.3%)	5 (19.2%)	17 (12.9%)	0 (0.0%)	**-**

SAPS II, Simplified Acute Physiological Score II; SOFA, Sequential Organ Failure Assessment score; D, Day; MV, mechanical ventilation; ICU, Intensive Care Unit; GCS, Glasgow coma scale; IAI, Intensive Care Unit associated Infection; p., p-value. Data are presented as numbers and percentages (qualitative variables) and medians and 25th/75th percentiles (quantitative variables). Categorical data were summarized by sample sizes and percentages. Cohorts were compared either with analysis of variance (ANOVA) test in case of normally distributed data or with Kruskal Wallis test by ranks for continuous data, and Chi-squared test or Fisher’s exact test, where required, for categorical data. No comparison were done for ‘D30 Urine Catheter free days’, ‘D30 Venous Catheter free days’, ‘D30 Intubation free days’, ‘D30 Hospital free days’, ‘D30 ICU free days’, ‘D28 Death’ and ‘D28 IAI’ as per these data require survival models for comparison (displayed by dash in “p.” column).

## Discussion

This study aimed at providing an improved understanding of the longitudinal modulation of mHLA-DR in sepsis patients during the first week after ICU admission and the association of observed changes in mHLA-DR with clinical characteristics and outcomes. Groups of patient that share a common mean trend in mHLA-DR expression over time were defined as endotypes based on the assumption that patients sharing similar mHLA-DR trajectories also share a common underlying biological mechanism (26). A common challenge in classification of patients into endotypes in prior studies is the lack of reproducibility. In the current study, we addressed this issue by including two independent cohorts to highlight common patterns of mHLA-DR evolution using *de novo* unsupervised clustering.

### Identifying Reliable Endotypes

In the Leijte et al. study that aimed at describing longitudinal mHLA-DR expression, a group-based trajectory model approach was applied to 109 patient trajectories (18). This method did not take into consideration missing data, required parameter measurements to be summarized over day groups (i.e. D1-D2, D3-D4 and D5-D7) and only included patients with samples in all these grouped days, thus leading to a patient selection bias. Our longitudinal clustering approach, KmL, uses imputation to handle missing data resulting in a higher precision to capture the evolution of a marker. This setting is therefore more accurate, does not bias the analyzed trajectories (27) and enables inclusion of all patients regardless of missing time points, which thus fits better in a clinical study context where some time points may be missing. Leijte et al. highlighted three endotypes: Non-improvers, Decliners, Improvers and an “Outlier” group. In our current study, with a much larger cohort, we confirmed these observations and extended them by defining a previously unreported mHLA-DR pattern, the High expressors endotype; this lack of previous reporting in the Leijte et al. study is likely due to the small number of patients and/or selection bias.

### Outcomes Characterization

Worse outcomes in the different mHLA-DR endotypes could help clinicians determine prognosis and potentially lead to endotype-specific immunotherapy (28–30). The current study demonstrated that length of ICU stay, IAI and death were not significantly different between patients characterized by the Non-improvers or Decliners endotypes. However, both of these endotypes were associated with worse outcomes (longer length of ICU stay and exposure to invasive devices, increased death and ICU Acquired Infections) compared to the Improvers and High expressors endotypes. Overall, 59% of sepsis patients were at risk of deterioration due to low surface mHLA-DR. It is postulated that the pathophysiology of these patients reflected persistent post-sepsis immunosuppression and these patients may have benefited from immunotherapy (31, 32).

### Additional Insights

An association between mHLA-DR and death and IAI occurrence has been previously described (18). However, the association of mHLA-DR trajectory with speed of recovery is reported here for the first time. The trends in mHLA-DR levels in this study show that approximately 90% of patients with the High expressor endotype were discharged from ICU at D11 versus only 40% with the Non-improvers endotype. This was confirmed over time with a sub-distribution HR of 4 for High expressors and 2 for Improvers in comparison to Non-improvers. SOFA, age and comorbidities (McCabe and Charlson score) were similar across endotypes at inclusion, hence suggesting that these characteristics did not explain the differences in mHLA-DR evolution over time. Of note, the sepsis infection site seemed to be associated with the Non-improvers endotype with approximately 28% of patients had a respiratory related infection; a worse outcome in pulmonary-sepsis compared to abdominal-sepsis has been previously described (33).

Longitudinal measurement of immune markers used for endotype definition enables the understanding of the evolution of a marker. Understanding the trajectory of a biomarker helps to highlight the minimal number of samples that is required in order to be able to assign accurately a patient to a specific endotype. In the context of mHLA-DR, this study has demonstrated that having D1 and D3 sampled for a patient are necessary for an early assignment to an endotype. Indeed, at D1 some of the Non-Improvers and Improvers have the same mHLA-DR level and are indistinguishable, and similarly, at D3 Decliners and High expressors mHLA-DR levels intersect. Further investigation is needed to understand the utility of a rapid assignment of a sepsis patient to an endotype and whether this may enable early treatment of patients to reduce the risk of clinical deterioration.

### Limitation

There are several limitations to the current study. Firstly, the longitudinal measurement of immune parameters over time assumes that the chosen time points are aligned across patients. However, as sepsis patients may have been identified at different times after the actual onset of sepsis and hence admitted to ICU at different times, this could have resulted in a shift in the mHLA-DR trajectories. This could explain the existence of the Decliners endotype in which patients could have been admitted earlier to the ICU in comparison to patients in the Non-improvers endotype, thus explaining why there was the same outcome for both endotypes. A further limitation of the study is that the optimum number of partitions in a given dataset still remains an unanswered question in the field of clustering; KmL method developers suggest the use of Calinski-Harabasz to set this point without *a priori* knowledge. In the present study, we decided to set the partition number to four following previous work in sepsis and it is unclear whether future studies using different numbers of partitions will result in the same conclusions.

## Conclusion

This study has defined four endotypes of mHLA-DR trajectories in septic patients and has demonstrated that the persistent monocyte deactivation during the first week of ICU admission is associated with increased ICU acquired infection, increased mortality and increase in both ICU and hospital length of stay. These findings indicate that this dysregulation is independent of patient clinical characteristics at admission and thus support the use of a biomarker, such as mHLA-DR measured over the first week, to help characterize patients. As mHLA-DR only reflects one aspect of the immune response, further studies evaluating the concomitant evolution of other immune markers will enable a more comprehensive characterization of the sepsis immune response. In conclusion, this study introduces a new method for defining clinical marker trajectory endotypes that can be used to better understand sepsis pathophysiology and paves the way towards future patient-adapted immunotherapy.

## REALISM Study Group

− HCL: Sophie ARNAL, Caroline AUGRIS-MATHIEU, Frédérique BAYLE, Liana CARUSO, Charles-Eric BER, Asma BEN-AMOR, Anne-Sophie BELLOCQ, Farida BENATIR, Anne BERTIN-MAGHIT, Marc BERTIN-MAGHIT, André BOIBIEUX, Yves BOUFFARD, Jean-Christophe CEJKA, Valérie CERRO, Jullien CROZON-CLAUZEL, Julien DAVIDSON, Sophie DEBORD-PEGUET, Benjamin DELWARDE, Robert DELEAT-BESSON, Claire DELSUC, Bertrand DEVIGNE, Laure FAYOLLE-PIVOT, Alexandre FAURE, Bernard FLOCCARD, Julie GATEL, Charline GENIN, Thibaut GIRARDOT, Arnaud GREGOIRE, Baptiste HENGY, Laetitia HURIAUX, Catherine JADAUD, Alain LEPAPE, Véronique LERAY, Anne-Claire LUKASZEWICZ, Guillaume MARCOTTE, Olivier MARTIN, Marie MATRAY, Delphine MAUCORT-BOULCH, Pascal MEURET, Céline MONARD, Florent MORICEAU, Guillaume MONNERET, Nathalie PANEL, Najia RAHALI, Thomas RIMMELE, Cyrille TRUC, Thomas UBERTI, Hélène VALLIN, Fabienne VENET, Sylvie TISSOT, Abbès ZADAM
− bioMérieux: Sophie BLEIN, Karen BRENGEL-PESCE, Elisabeth CERRATO, Valérie CHEYNET, Emmanuelle GALLET-GORIUS, Audrey GUICHARD, Camille JOURDAN, Natacha KOENIG, François MALLET, Boris MEUNIER, Virginie MOUCADEL, Marine MOMMERT, Guy ORIOL, Alexandre PACHOT, Estelle PERONNET, Claire SCHREVEL, Olivier TABONE, Julien TEXTORIS, Javier YUGUEROS MARCOS
− BIOASTER: Jérémie BECKER, Frédéric BEQUET, Yacine BOUNAB, Florian BRAJON, Bertrand CANARD, Muriel COLLUS, Nathalie GARCON, Irène GORSE, Cyril GUYARD, Fabien LAVOCAT, Philippe LEISSNER, Karen LOUIS, Maxime MISTRETTA, Jeanne MORINIERE, Yoann MOUSCAZ, Laura NOAILLES, Magali PERRET, Frédéric REYNIER, Cindy RIFFAUD, Mary-Luz ROL, Nicolas SAPAY, Trang TRAN, Christophe VEDRINE
− SANOFI: Christophe CARRE, Pierre CORTEZ, Aymeric DE MONFORT, Karine FLORIN, Laurent FRAISSE, Isabelle FUGIER, Sandrine PAYRARD, Annick PELERAUX, Laurence QUEMENEUR
− ESPCI: Andrew GRIFFITHS, Stephanie TOETSCH
− GSK: Teri ASHTON, Peter J. GOUGH, Scott B. BERGER, David GARDINER, Iain GILLESPIE, Aidan MACNAMARA, Aparna RAYCHAUDHURI, Rob SMYLIE, Lionel TAN, Craig TIPPLE

## Data Availability Statement

The original contributions presented in the study are included in the article/**Supplementary Material**, further inquiries can be directed to karen.brengel-pesce@biomerieux.com.

## Ethics Statement

The IMMUNOSEPSIS cohort is registered with the French Ministry of Research and Teaching (#DC-2008-509), at the Commission Nationale de l’Informatique et des Libertés, at Clinicaltrials.gov (NCT02803346) and has been approved by the Institutional Study Board (IRB11236). The REALISM study is registered at ClinicalTrials.gov (NCT02638779) and have been approved by the Institutional Study Board (2015-42-2). The patients/participants provided their written informed consent to participate in this study.

## Author Contributions

GM, DM-B, EP, and MB conceived the study. MB performed biostatistic and bioinformatic analysis of the data. GM, DM-B, EP, and MB interpreted the results. GM, DM-B, EP, and MB drafted the manuscript. All authors were involved in the revision of the manuscript and approved the final version.

## Funding

REALISM study received funding from the Agence Nationale de la Recherche through a grant awarded to BIOASTER (Grant number #ANR-10-AIRT-03) and from bioMérieux, Sanofi and GSK. The Agence Nationale de la Recherche was not involved in the study design, collection, analysis, interpretation of data, the writing of this article or the decision to submit it for publication. IMMUNOSEPSIS study received funding from Hospices Civils de Lyon which was involved in the IMMUNOSEPSIS study design, collection, analysis, interpretation of data, writing of report, and decision to submit the article for publication. MB was supported by the Association Nationale de la Recherche et de la Technologie (ANRT), convention N° 2020/0371.

## Conflict of Interest

MB, JT, KB-P, and EP are employees of bioMérieux SA, an *in vitro* diagnostic company. FC, TR, FV, GM, and DM-B are employees of Hospices Civils de Lyon. MB, EP, KB-P, JT, TR, FC, and GM work in a joint research unit, co funded by the Hospices Civils de Lyon and bioMérieux. LT is employee of and holds stock and shares in GlaxoSmithKline. LQ is an employee of Sanofi Pasteur. CV is employee of BIOASTER. AG is employee of ESPCI Paris.

The authors declare this study received funding from bioMérieux Sanofi and GSK. The funders were involved in the REALISM study design, collection, analysis, interpretation of data, writing of report, and decision to submit the article for publication.

## Publisher’s Note

All claims expressed in this article are solely those of the authors and do not necessarily represent those of their affiliated organizations, or those of the publisher, the editors and the reviewers. Any product that may be evaluated in this article, or claim that may be made by its manufacturer, is not guaranteed or endorsed by the publisher.

## Abbreviations

mHLA-DR, Count of antibodies linked to monocyte’s Human Leukocyte Antigen – DR isotype (mHLA-DR) surface proteins; AB/C, Antibody per cells; ICU, Intensive Care Unit; IAI, ICU Acquired Infection; RRT, Renal Replacement Therapy; GCS, Glasgow Coma Scale; SAPS II, Simplified Acute Physiology Score; SOFA, Sequential Organ Failure Assessment; sHR, subdistribution Hazard Ratio; CI, Confidence Interval; KmL, K-means for longitudinal data.
