# Supplementary material for: Monocyte Trajectories Endotypes Are Associated With Worsening in Septic Patients
Source: Front Immunol. 2021 Nov 29;12:795052. doi: 10.3389/fimmu.2021.795052 (PMC8667763; doi:10.3389/fimmu.2021.795052)
Supplement: Supplementary file 1 [file DataSheet_1.pdf]

SUPPLEMENTARY MATERIALS

Bodinier *et al.*, Monocyte trajectories endotypes are associated with worsening in septic patients.

doi: 10.3389/fimmu.2021.795052

SUPPLEMENTARY FIGURES

sFigure 1. Analysis workflow

“N” is the initial number of patients and “n” is the number of patients after exclusion of outlier samples (Tukey method) and patients with only one sample. In the Discovery cohort, 8 outlier samples were excluded along with 54 patients due to unique time point resulting in a total of 276 patients included. In Verification cohort, 4 outlier samples were excluded along with 4 patients due to unique time point resulting in a total of 102 patients included.

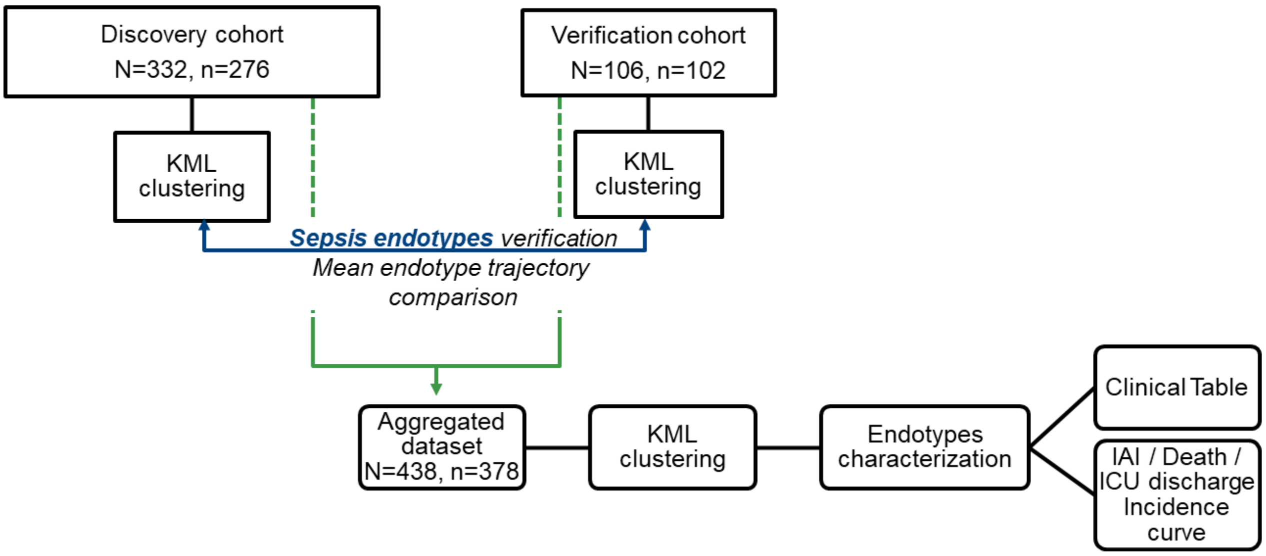

sFigure 2. Endotypes matching between Aggregated and Discovery / Verification cohorts.

Endotypes observed after a *de novo* clustering on Aggregated dataset (Discovery and Verification cohorts) clustering have been matched with ones obtained in Discovery only and Validation only cohorts. Cohorts are represented in x-axis, with bars. Each bar is subdivided by endotype. y-axis depicts number of patients. Patients flow between endotypes is represented by curve, either red when the endotype is not matching, either green when it is matching.

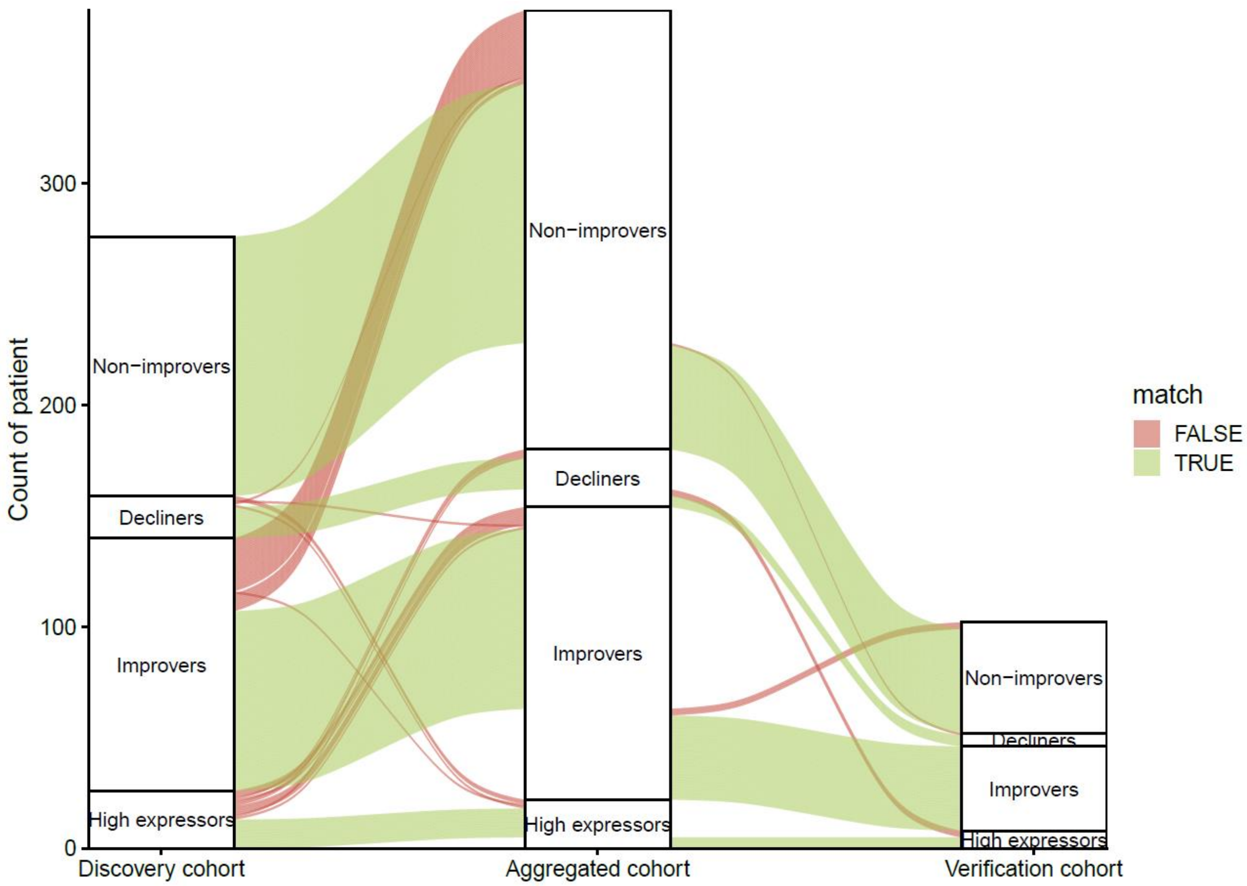

doi: 10.3389/fimmu.2021.795052

sFigure 3. Repartition of endotypes across the different combinations of time points in the Aggregated dataset.

The different time points (black dots) combinations of aggregated dataset patients are represented in column along with marginal counts as histograms (left: count of patients sampled for the time point and top: count of patients having the combination of time points). To graphically observe the repartition of endotypes across these combination, top histogram's bars are colored: "Non-improvers" (red), "Decliners" (yellow), "Improvers" (light green) and "High expressors" (green).

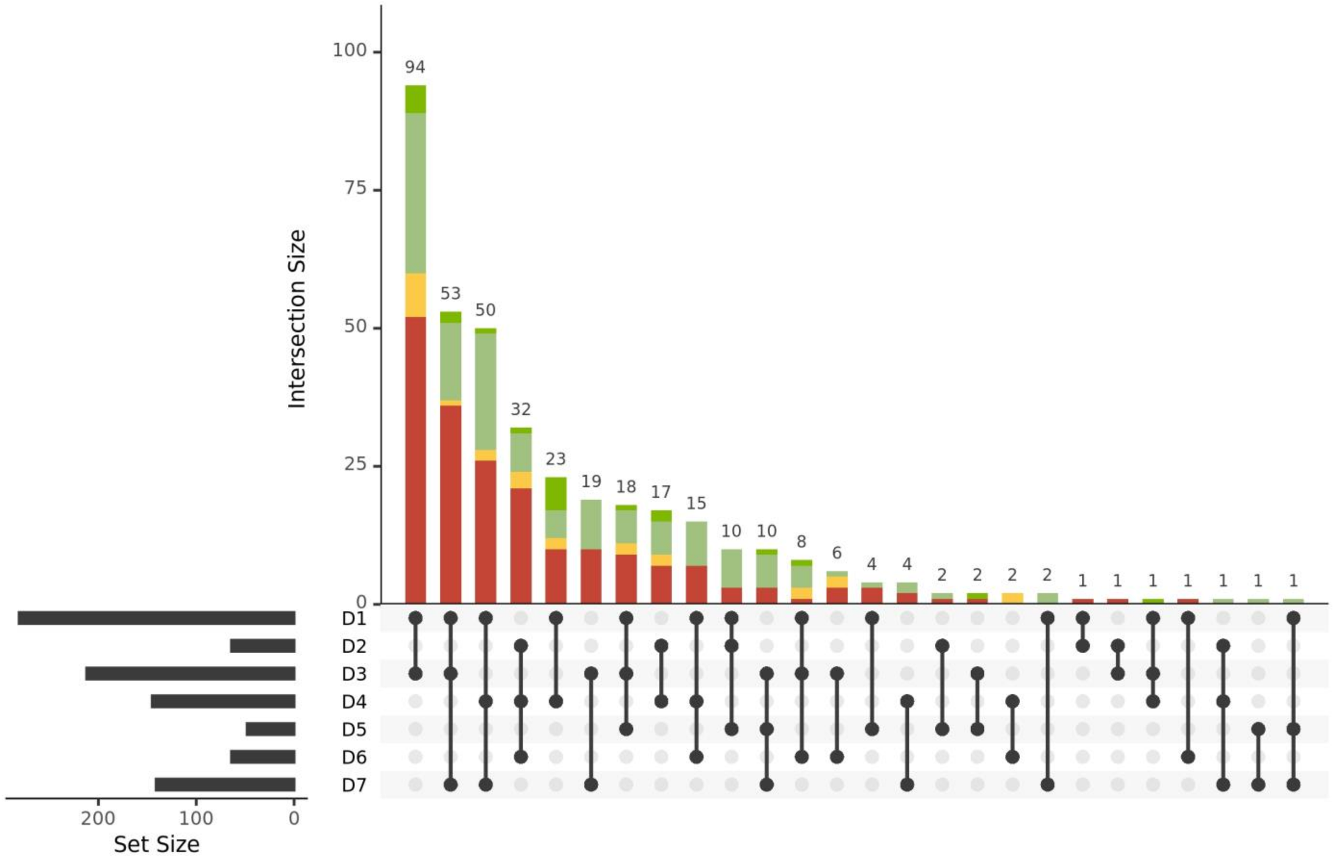

doi: 10.3389/fimmu.2021.795052

SUPPLEMENTARY TABLES

sTable1. Classification quality among cohorts

Patient's probabilities to belong to one of the four clusters (column) has been summarized as the mean by assigned cluster (row). Cluster 1: Non-improvers, Cluster 2: Decliners, Cluster 3: Improvers, Cluster 4: High improvers.

A: DISCOVERY COHORT

	Mean probability			
Assigned Cluster	**Cluster 1**	**Cluster 2**	**Cluster 3**	**Cluster 4**
**1**	**0.823**	0.000	0.175	0.002
**2**	0.000	**0.924**	0.013	0.062
**3**	0.206	0.001	**0.787**	0.006
**4**	0.004	0.006	0.132	**0.858**

B: VERIFICATION COHORT

	Mean probability			
Assigned Cluster	**Cluster 1**	**Cluster 2**	**Cluster 3**	**Cluster 4**
**1**	**0.916**	0.007	0.077	0.000
**2**	0.131	**0.824**	0.037	0.008
**3**	0.155	0.004	**0.841**	0.000
**4**	0.000	0.091	0.121	**0.788**

C: SEPSIS COHORT

	Mean probability			
Assigned Cluster	**Cluster 1**	**Cluster 2**	**Cluster 3**	**Cluster 4**
**1**	**0.877**	0.000	0.123	0.000
**2**	0.002	**0.889**	0.093	0.015
**3**	0.241	0.002	**0.755**	0.001
**4**	0.001	0.008	0.133	**0.858**

doi: 10.3389/fimmu.2021.795052

sTable 2. Mean trajectories differences between discovery and verification cohorts.

A mixed model comparing boxcoxed mHLA-DR trajectories endotypes across discovery and verification dataset was run. Fixed effect follow a time second degree polynomial equation and random effect a time linear equation. The fixed effect equation is as follow: $mHLA - DR = a \times time^2 + b \times time + c$. The table below reports parameters *a*, *b* and *c* (rows) along with the p values of a t-test comparing these parameters between cohorts (columns).

	**Non-improvers Endotype**			**Decliners Endotype**			**Improvers Endotype**			**High expressors Endotype**		
	**Discovery**	**Verification**	**p.**	**Discovery**	**Verification**	**p.**	**Discovery**	**Verification**	**p.**	**Discovery**	**Verification**	**p.**
**a**	0.0129	0.0093	0.3209	0.0237	-0.0020	0.3396	0.0258	0.0317	0.2717	0.0027	-0.0089	0.2918
**b**	-0.0784	-0.0592	0.4893	-0.2781	-0.1571	0.1695	0.0268	-0.0060	0.4353	-0.0042	0.1330	0.1137
**c**	5.0847	5.0320	0.273	5.9664	5.8307	0.3738	5.2294	5.2076	0.7656	5.5660	5.1822	0.0132

doi: 10.3389/fimmu.2021.795052

sTable 3. Mean mHLA-DR value at each time point in the aggregated dataset.

Mean mHLA-DR values (AB/C) from a linear mixed model fitted to aggregated endotypes were reported here along with CI 95% intervals in parenthesis.

	D1	D2	D3	D4	D5	D6	D7
**Non-improvers n=198**	3265<br>(3113-3430)	3038<br>(2934-3149)	2980<br>(2875-3095)	3078<br>(2963-3202)	3358<br>(3225-3490)	3865<br>(3698-4038)	4706<br>(4426-5045)
**Decliners n=26**	31774<br>(26365-38546)	19102<br>(16933-21747)	13614<br>(12031-15339)	11118<br>(9700-12497)	10281<br>(8997-11619)	10785<br>(9096-12605)	12926<br>(9758-16496)
**Improvers n=132**	6331<br>(5936-6780)	6806<br>(6495-7166)	7386<br>(7008-7743)	8054<br>(7621-8467)	8838<br>(8364-9297)	9787<br>(9210-10381)	10921<br>(10000-11875)
**High expressors n=22**	9681<br>(8216-11357)	12477<br>(10996-14172)	15821<br>(13938-17978)	19739<br>(17126-22782)	24146<br>(20401-28062)	28604<br>(22851-36186)	33334<br>(22983-48422)

doi: 10.3389/fimmu.2021.795052
